# Dataset of antibody variable region sequence features inferred from a respiratory syncytial virus fusion protein-specific B cell receptor repertoire induced by natural infection of a healthy adult

**DOI:** 10.1016/j.dib.2020.106499

**Published:** 2020-11-04

**Authors:** Gerald Schneikart, Simona Tavarini, Chiara Sammicheli, Giulia Torricelli, Silvia Guidotti, Ugo D'Oro, Oretta Finco, Monia Bardelli

**Affiliations:** a GSK, Via Fiorentina 1, 53100 Siena, Italy; b Department of Life Sciences, University of Siena, Via Aldo Moro 2, 53100 Siena, Italy

**Keywords:** Antibody repertoire analysis, B cell receptor repertoire, Clonal expansion, Clonal relatedness, Memory B cell, Respiratory syncytial virus, Fusion protein, Natural infection

## Abstract

Respiratory syncytial virus (RSV) is the primary cause for acute lower respiratory syndrome in children younger than 5 years. Research on B cell repertoires and antibodies binding the RSV fusion protein (RSV F) is of major interest in the development of potential vaccine candidates and therapies. B cell receptors (BCRs) which have higher affinities for a specific antigen are preferentially selected for B cell clonal expansion in germinal center reactions. Consequently, antigen-specific BCR repertoires share common features, as for instance preferential variable gene usage, variable region mutation levels or lengths of the heavy chain complementarity-determining region 3. Since RSV repeatedly infects every person throughout life, memory B cells (MBC) expressing RSV F-binding BCRs circulate in the blood of healthy adults. This dataset of BCR variable region sequence features was derived from single cell-sorted RSV F-directed MBCs of a healthy adult blood donor [1]. The dataset was produced with publicly available data analysis software programs and scripts, which facilitates integration or comparison with antibody sequence repertoire data of different individuals derived with the same or comparable data analysis approaches and tools.

**Specifications Table**

**Table:** 

Subject	Immunology
Specific subject area	Respiratory syncytial virus fusion protein-specific B cell receptor repertoires
Type of data	Table Image Chart Graph Figure
How data were acquired	Cloanalyst (available from: http://www.bu.edu/computationalimmunology/research/software/) for the implementation of a Bayesian method [2, 3]; Bayesian estimation of Antigen-driven SELectIoN (BASELINe version 1.3; R script: http://selection.med.yale.edu/baseline/Archive/) [4]
Data format	Raw Analyzed Filtered
Parameters for data collection	B cell receptor sequences represented in FASTA format served as input for analyses. Before BASELINe analyses using standard settings (Species: Human; substitution model: S5F [5]; Mutability Model: S5F [5]; Clonal: Independent sequences; fix Indels: Do nothing), sequences were further reformatted according to the IMGT unique numbering system [6].
Description of data collection	B cell receptor sequences were isolated from single cell-sorted RSV fusion protein-binding memory B cells [1]. The datasets of B cell receptor V and J gene usages, V region mutations, CDR3 lengths and clonal relatedness were produced by the implementation of a Bayesian method (Cloanalyst, available from: http://www.bu.edu/computationalimmunology/research/software/) [2, 3]. The datasets of selection strengths on replacement mutations were derived with the BASELINe script for R (version 1.3: http://selection.med.yale.edu/baseline/Archive/) [4].
Data source location	GSK, Siena, Italy
Data accessibility	With the article
Related research article	G. Schneikart, S. Tavarini, C. Sammicheli, G. Torricelli, S. Guidotti, E. Andreano, F. Buricchi, U. D'Oro, O. Finco, M. Bardelli, The respiratory syncytial virus fusion protein-specific B cell receptor repertoire reshaped by post-fusion subunit vaccination, 2020, Vaccine. In Press.

## Value of the Data

• The data enables characterization of a memory B cell receptor (BCR) repertoire directed against the fusion protein of RSV.
• The data can be used in different studies on RSV F-specific BCR repertoires. For example, comparison of the dataset with vaccine-induced RSV F-directed BCR repertoires may provide insights on how a certain vaccine reshapes RSV F-binding BCR repertoires, which were previously induced by natural infections.
• The data can be integrated and compared with any dataset of BCR sequence features, which was derived with the same or comparable means of antibody sequence analysis.
• The methods applied to acquire this dataset are applicable for BCR repertoire analyses in the context of different antigens and pathogens.

**Fig. 1:**
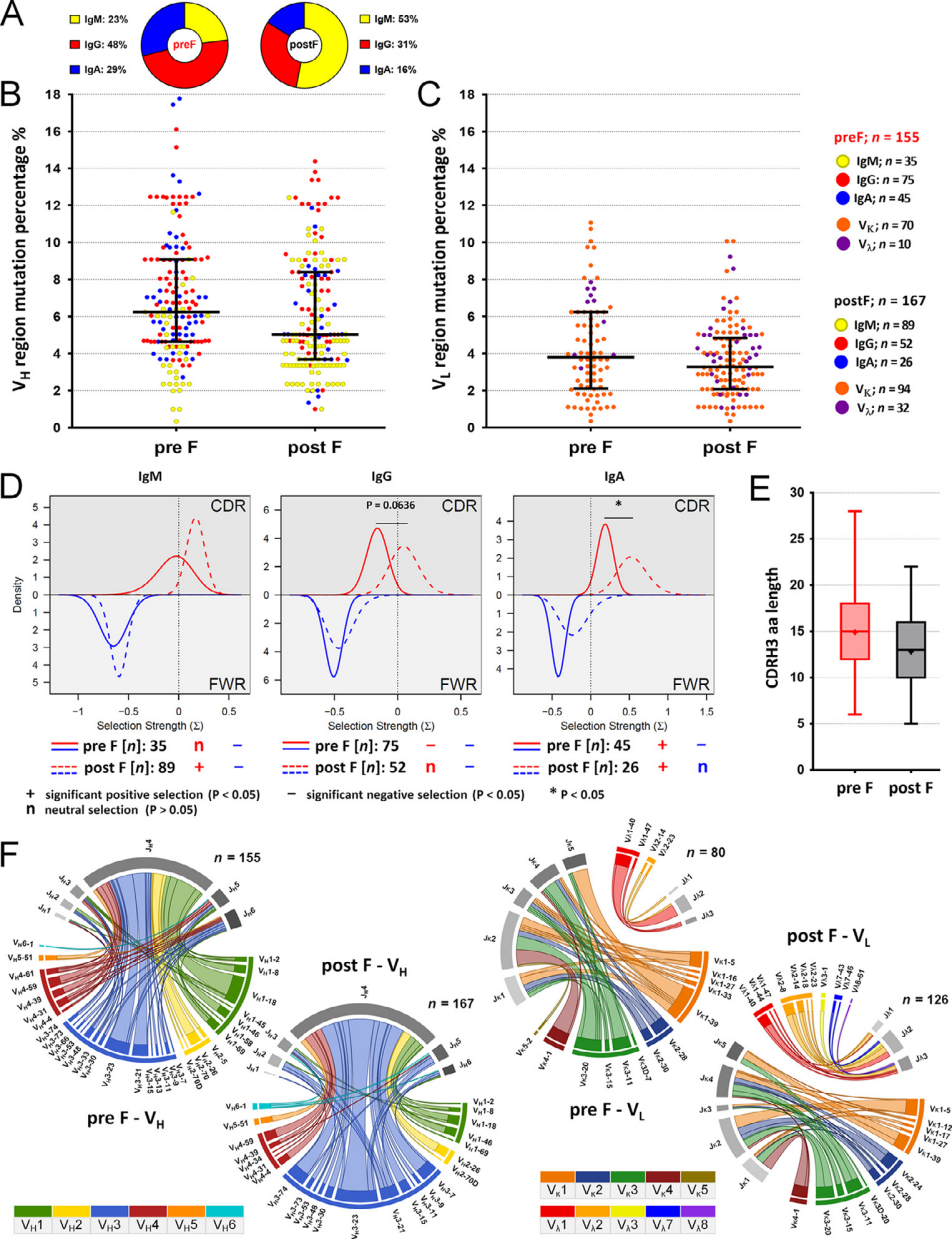
Characteristics of the pre F- or post F-isolated BCR repertoires induced by natural RSV-infection. The color legend on the top right lists the numbers of analyzed pre F- or post F-isolated IgM, IgG, IgA, V_κ_ or V_λ_ sequences (*n*). **(A)** Relative frequencies (%) of IgM (yellow), IgG (red) and IgA (blue) isotypes in the pre F- (left) and post F-isolated (right) BCR repertoires. **(B)** V_H_ region mutation percentages of pre F- or post F-isolated IgMs (yellow), IgGs (red) and IgAs (blue). The medians and the inter-quartile ranges are indicated. **(C)** V_L_ region mutation percentages of pre F- or post F-isolated V_κ_ (orange) or V_λ_ (magenta) sequences. The medians and the inter-quartile ranges are indicated. **(D)** Posterior probability density functions of selection strengths (Σ) on replacement mutations in the CDRHs (upper halves) and the FWRHs (bottom halves) of pre F- (continuous) or post F-isolated (dashed) IgMs (left), IgGs (middle) or IgAs (right). Statistical significances of Σ-values indicating positive (*P* < 0.05), negative (*P* < 0.05) or neutral selections (*P* > 0.05) are signified by the symbols “+”, “-“ or “n”, respectively. The asterisk indicates a statistically significant difference between the selection strengths in pre F- and post F-isolated CDRHs. **(E)** CDRH3 amino acid length distributions in the pre F- (red) or post F-isolated (black) BCR repertoires. Boxes show locations of 25, 50 and 75 percentiles, while whiskers reach to minimum and maximum values. Mean values are indicated by “+”. **(F)** The CIRCOS plots illustrate rearrangements of V_H_ and J_H_ or V_L_ and J_L_ genes in the pre F- and post F-isolated BCR repertoires. The lengths of each arc correspond to the relative frequencies of V or J gene segments used, while the widths of each ribbon demonstrate their relative connection frequencies. Arc colors were assigned for each V and J gene family used, as indicated in the color legend. Ribbons have the same colors as the connected V-gene segments. *n*: numbers of analyzed sequences.

## 1 Data Description

B cell receptor (BCR) sequences binding the pre-fusion (pre F) or post-fusion (post F) conformation of RSV F were derived from single cell-sorted memory B cells (MBCs) of the healthy adult blood donor BD09 [1]. The dataset of variable region (V_H_, V_κ_ and V_λ_) sequence features characterizing each single BCR in the repertoire was acquired using the bioinformatics software tools described in the methods sections, and is provided in tabular form in the Supplementary Table 1 (V_H_), Supplementary Table 2 (V_κ_), and Supplementary Table 3 (V_λ_). The isotypes of each BCR, which are listed in the Supplementary Table 4 (isotypes), were identified by the first four codons of the constant regions [7].

The numbers of analyzed pre F- and post F-specific IgM, IgG, IgA, V_κ_ or V_λ_ sequences are listed in the color legend on the top right in Fig. 1. Fig. 1A summarizes the relative frequencies (%) of IgM, IgG and IgA isotypes in the pre F- or post F-isolated BCR repertoires. Fig. 1B shows the distributions of V_H_ region mutation percentages of pre F- or post F-isolated IgMs, IgGs and IgAs, while Fig. 1C depicts the distributions of V_L_ region mutation percentages of pre F- or post F-isolated V_κ_ or V_λ_ sequences.

The posterior probability density functions of selection strengths (Σ) on replacement mutations in the heavy chain complementarity-determining regions (CDRH) and the heavy chain framework regions (FWRH) of the pre F- or post F-isolated IgMs, IgGs or IgAs are visualized in Fig. 1D. Calculations were performed with BASELINe, version 1.3 [4], which is described in the methods section. Pre F- and post F-binding IgM, IgG, or IgA sequences were pooled and grouped into the categories ‘post F.IgM’ and ‘pre F.IgM’, ‘post F.IgG’ and ‘pre F.IgG’, or ‘post F.IgA’ and ‘pre F.IgA’, respectively. The BASELINe analysis output data, including the calculations of the Σ-values, the plots of the posterior probability distribution functions of Σ, and the statistical comparisons (*p*-values), are provided in the Supplementary Data (Supplementary Tables 5, 6, and 7, and PDF-files).

The CDRH3 amino acid length distributions in the pre F or post F BCR repertoires are shown in Fig. 1E. The CIRCOS plots in Fig. 1F illustrate the rearrangements of V_H_ and J_H_ or V_L_ and J_L_ genes in the pre F- or post F-isolated BCR repertoires.

**Fig. 2:**
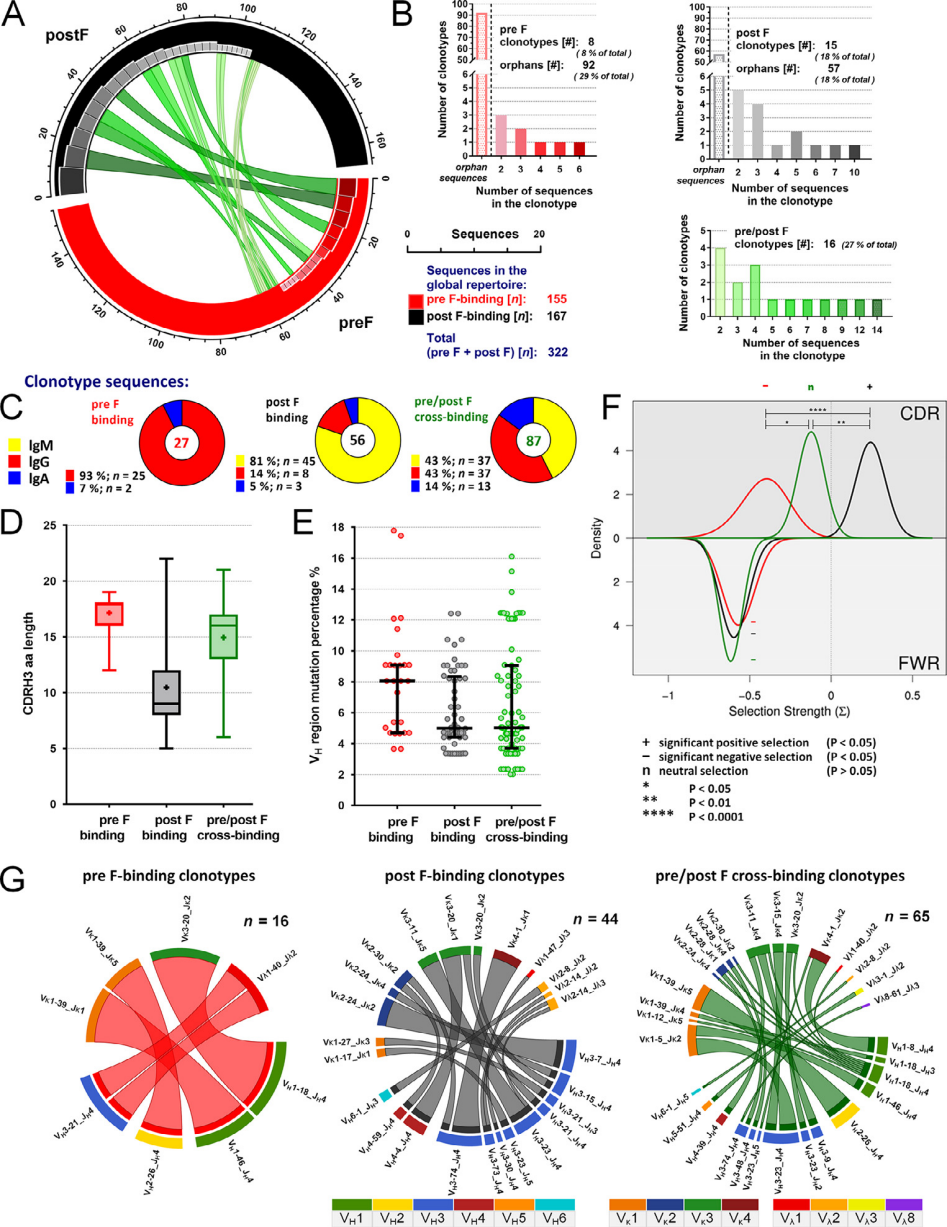
Characteristics of clonally related pre F-, post F- and pre/post F cross-binding BCRs. **(A)** The CIRCOS plot illustrates the clonal relatedness in the pre (red arc) and post (black arc) F-isolated BCR repertoires. Positions on the arcs are occupied by single BCR sequences, as indicated by the ruler. Arc lengths correspond to numbers of analyzed sequences (*n*). Red or gray rectangles illustrate pre F- or post F-binding clonotypes (clonally related BCR sequences isolated with pre F or post F protein), respectively, while green bands connect clonally related pre F- and post F-isolated BCR sequences (pre/post F cross-binding clonotypes). Rectangle or band widths and color shadings (light to dark) are associated with certain numbers of BCR sequences in single clonotypes. **(B)** The bar charts summarize the amount of estimated pre F-, post F- or pre/post F cross-binding clonotypes having different numbers of BCR sequences. Bar heights correlate with the amount of clonotypes containing a certain number of BCR sequences (X-axis); color shadings for clonotype sizes is the same as for rectangles and bands in the CIRCOS plot. Numbers (#) and relative frequencies (% of 322 total BCRs) of pre F-, post F- or pre/post F cross-binding clonotypes or orphan sequences (which did not group with other BCR sequences) are indicated on top of each chart. **(C)** Relative frequencies (%) of IgM (yellow), IgG (red) and IgA (blue) isotypes of pre F- (left), post F- (middle) or pre/post F cross-binding (right) clonotypes. **(D)** CDRH3 amino acid length distributions of pre F- (red), post F- (black) or pre/post F cross-binding (green) clonotypes. Boxes show locations of 25, 50 and 75 percentiles, while whiskers reach to minimum and maximum values. Mean values are indicated by “+”. **(E)** V_H_ region mutation percentages of pre F- (red), post F- (black) or pre/post F cross-binding (green) clonotypes. The medians and the inter-quartile ranges are indicated. **(F)** Posterior probability density functions of selection strengths (Σ) on replacement mutations in the CDRHs (upper half) and the FWRHs (bottom half) of pre F- (red), post F- (black) or pre/post F cross-binding (green) clonotypes. Pre F-, post F- or pre/post F cross-binding clonotype sequences were pooled and grouped before the analysis. Statistical significances of Σ-values indicating positive (*P* < 0.05), negative (*P* < 0.05) or neutral selections (*P* > 0.05) are signified by the symbols “+”, “-” or “n”, respectively. The bars indicate statistically significant differences between selection strengths and the asterisks refer to the respective significance levels as shown in the legend. **(G)** Combinations of heavy and light V-J gene rearrangements in pre F- (left), post F- (middle) or pre/post F cross-binding (right) clonotypes. The outer most arcs represent V-J gene rearrangements (lower arcs: V_H_; upper arcs: V_L_). Colors of V_H_, V_κ_ or V_λ_ families are shown in the legend. Additional sectors above the V_H_-J_H_ gene rearrangements correspond to single clonotypes using those V_H_-J_H_ gene rearrangements, while the sector lengths correlate with numbers of BCR sequences in the clonotype. The bands connect pairs of V_H_-J_H_|V_L_-J_L_ gene combinations. *n*: numbers of analyzed V_H_-V_L_ sequence pairs.

The clonal relatedness data of the pre F- or post F-isolated BCR repertoires is visualized in Fig. 2A. The data was acquired using a Bayesian method [2, 3], as described in the method section, and is included in the Supplementary Table 1 (the CloneIDs in the CloneAssignments-tabs indicate clonal relatedness between the isolated BCRs).

The bar charts in Fig. 2B summarize the amount of clonally related pre F- and post F-isolated BCR sequences. A group of clonally related BCR sequences is referred to as clonotype. Clonotypes, which consist of both pre F- and post F-isolated BCRs are indicated as pre/post F cross-binding clonotypes. Fig. 2C shows the relative frequencies (%) of IgM, IgG and IgA isotypes of pre F-, post F- or pre/post F cross-binding clonotypes. Fig. 2D depicts the CDRH3 amino acid length distributions of pre F-, post F- or pre/post F cross-binding clonotypes. Fig. 2E illustrates the distributions of V_H_ region mutation percentages of pre F-, post F- or pre/post F cross-binding clonotype sequences.

The plot in Fig. 2F graphs the posterior probability density functions of selection strengths (Σ) on replacement mutations in the CDRHs and the FWRHs of pre F-, post F- or pre/post F cross-binding clonotypes. Calculations were performed with BASELINe, version 1.3 [4], which is described in the methods section. Pre F-, post F- and pre/post F cross-binding clonotype sequences were pooled and grouped into the categories ‘pre F-clonotypes’, ‘post F-clonotypes’, and ‘pre F-post F-clonotypes’ before the analysis. The BASELINe output data, including the calculations of the Σ-values, the plots of the posterior probability distribution functions of Σ, and the statistical comparisons (*p*-values), are provided in the Supplementary Data (Supplementary Table 8 and PDF-files).

The CIRCOS plots in Fig. 2G illustrate the combinations of heavy and light V-J gene rearrangements in pre F-, post F- or pre/post F cross-binding clonotypes sequences, which belonged to completely recovered V_H_-V_L_ sequence pairs.

## 3 Experimental Design, Materials and Methods

After isolating RSV pre or post F-binding BCR sequences from single cell-sorted MBCs of the healthy blood donor BD09 [1], the datasets of BCR V and J gene usages, V region mutations, CDR3 lengths and clonal relatedness were acquired using the Cloanalyst software program (available from: http://www.bu.edu/computationalimmunology/research/software/) for implementation of a Bayesian method [2, 3]. Sequences with no identifiable CDR3 or one of the conserved amino acids missing (C23, W41 or C104; according to the unique IMGT numbering system [6]) were excluded from the analyses. The first four codons of the constant regions allowed the identification of isotypes [7].

Data on mutation selection strength was acquired using Bayesian estimation of Antigen-driven SELectIoN (BASELINe version 1.3; R script: http://selection.med.yale.edu/baseline/Archive/) to measure selection strengths on replacement mutations [4]. BASELINe detects mutations by comparing mutated sequences with their unmutated germline sequences and categorizes them in silent (S) or replacement (R) mutations in CDRs or FWRs (S_CDR_, R_CDR_, S_FWR_, R_FWR_). Then, it calculates expected mutation frequencies based on an underlying mutability model to account for hotspot and coldspot motifs [4, 5]. Afterwards, BASELINe derives posterior probability distribution functions of estimated observed mutation frequencies and compares them with expected frequencies in log-odds ratios to quantify selection strength (Σ). Positive Σ-values represent higher frequencies of replacement mutations than expected, which suggests positive selection. Negative Σ-values are the result of higher frequencies of silent mutations (no selection pressure on silent mutations) than replacement mutations, which indicates negative selection. BASELINe allows statistical comparisons of selections between independent sequences encoded by different germline genes or groups of sequences (repertoires) [4]. FASTA sequences reformatted according to the IMGT unique numbering system served as input for BASELINe [6], while the CDR3 and the FWR4 were excluded because of uncertainties in estimation of the D gene usage [2, 8]. Furthermore, sequences with insertions were also excluded from analyses.

BCR variable region sequence feature datasets were managed with Excel 2010 (Microsoft Corporation), and visualized with GraphPad Prism Version 7.00 (GraphPad Software) or R scripts (circlize package to produce CIRCOS plots: https://CRAN.R-project.org/package=circlize; BASELINe version 1.3 to graph posterior probability distribution functions: http://selection.med.yale.edu/baseline/Archive/).

## 4 Ethics Statement

A 50 ml blood sample of the healthy donor BD09, who signed an informed consent form, was contractually provided by the San Giuseppe hospital in Empoli, Tuscany, Italy. The study was approved by local ethic committees and conducted according to good clinical practice in accordance with the declaration of Helsinki (European Council 2001, US Code of Federal Regulations, ICH 1997).

## Declaration of Competing Interest

The authors have declared the following potential conflicts of interest: Simona Tavarini, Chiara Sammicheli, Silvia Guidotti, Giulia Torricelli, Ugo D'Oro, Oretta Finco and Monia Bardelli are employees of the GSK group of companies. Gerald Schneikart participated in a post-graduate studentship at GSK.

## Appendix Supplementary materials

Image, application 1
