# Supplementary figures and images for: Dataset of antibody variable region sequence features inferred from a respiratory syncytial virus fusion protein-specific B cell receptor repertoire induced by natural infection of a healthy adult
Source: Data Brief. 2020 Nov 4;33:106499. doi: 10.1016/j.dib.2020.106499 (PMC7666335; doi:10.1016/j.dib.2020.106499)

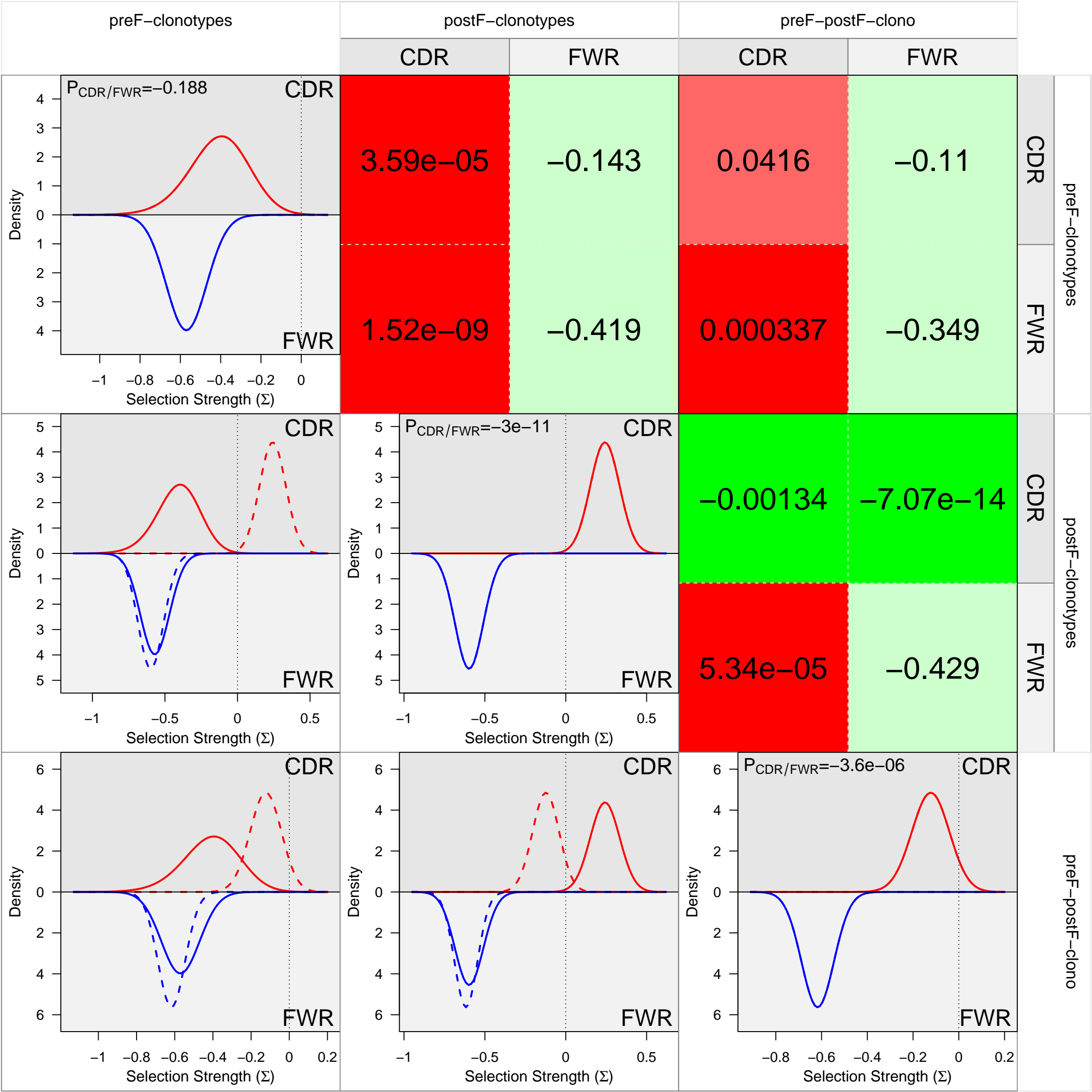

Supplement: Supplementary file 1 [file mmc1.zip › Supplementary_material/BD09.Clonotypes.pdf]

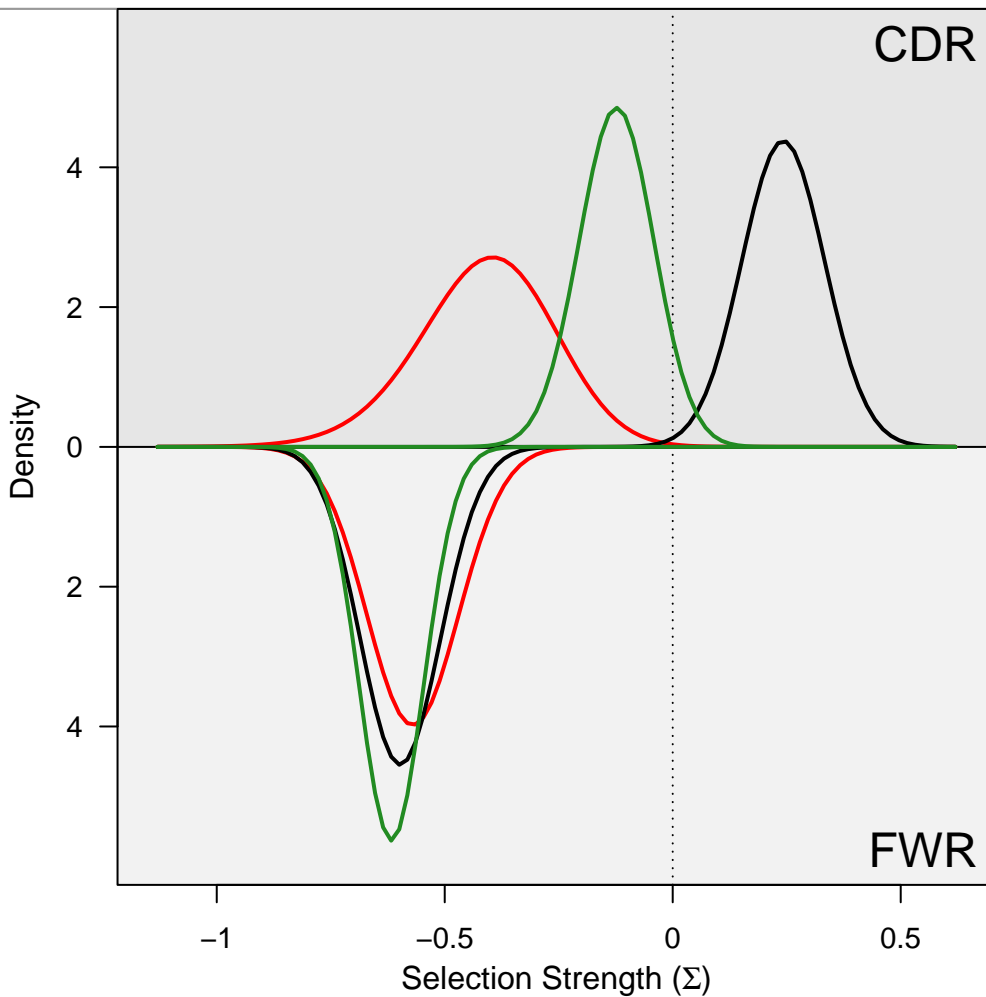

Supplement: Supplementary file 1 [file mmc1.zip › Supplementary_material/BD09.Clonotypes_comparison.pdf]

BD09.postF.IgA

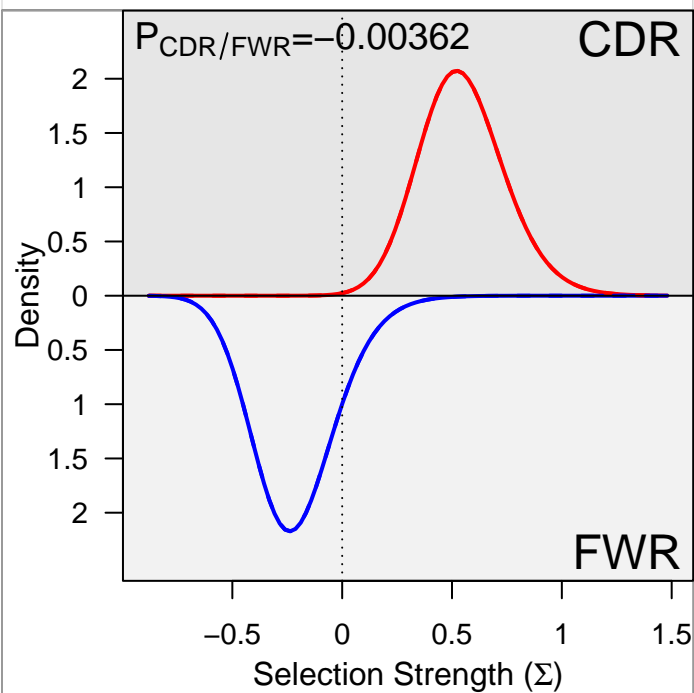

BD09.preF.IgA

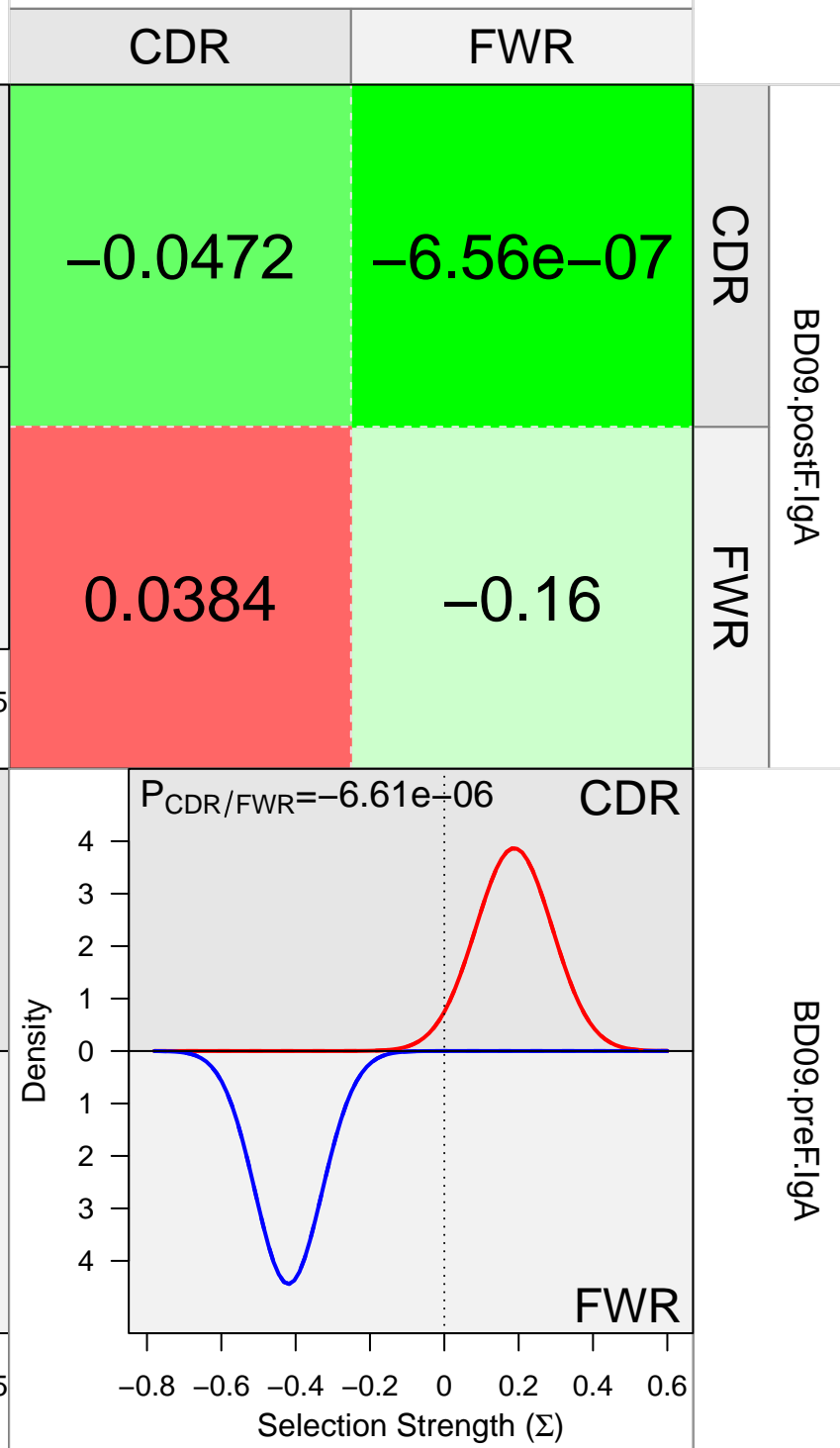

Supplement: Supplementary file 1 [file mmc1.zip › Supplementary_material/BD09.IgA.pdf]

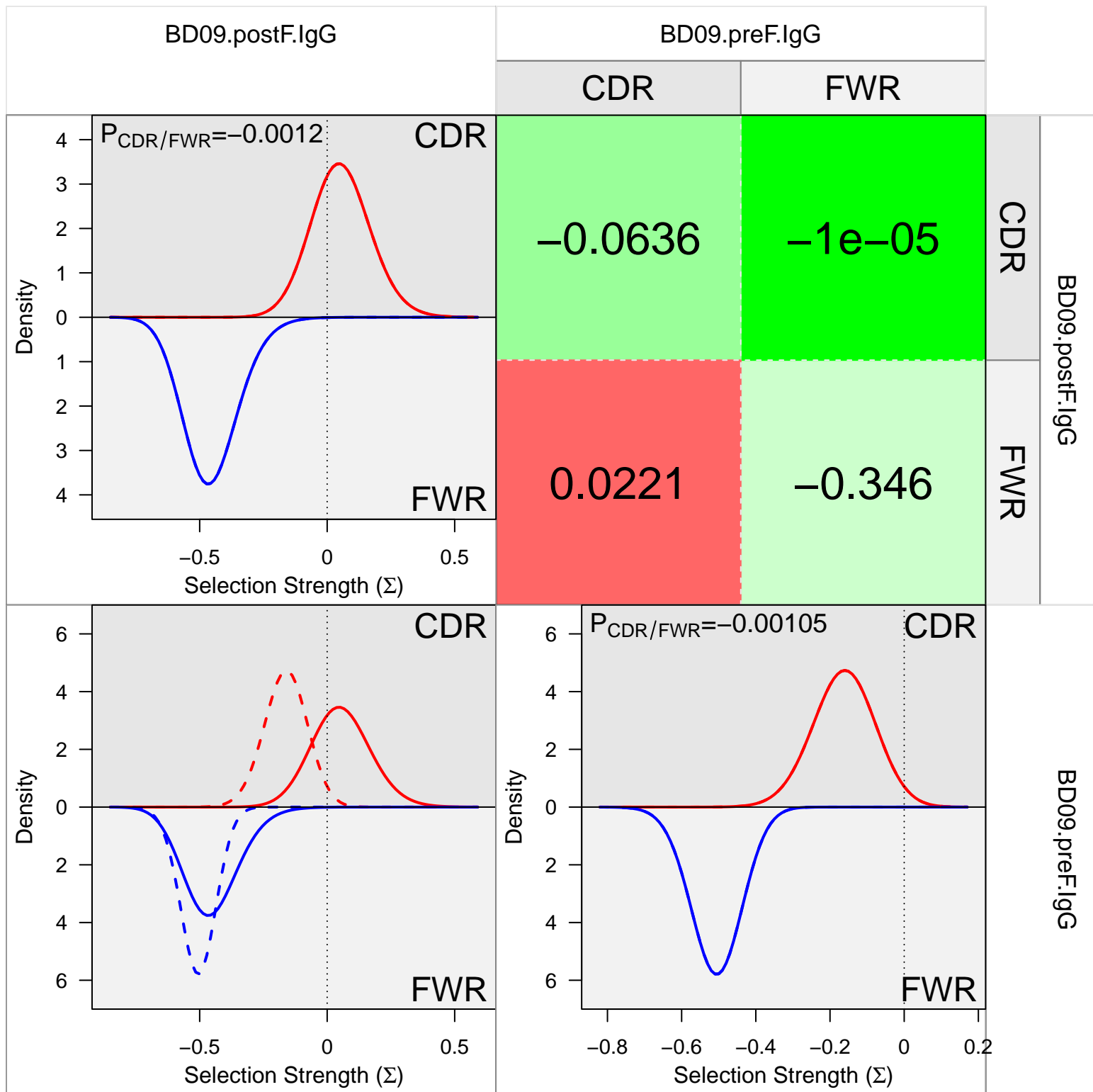

Supplement: Supplementary file 1 [file mmc1.zip › Supplementary_material/BD09.IgG.pdf]

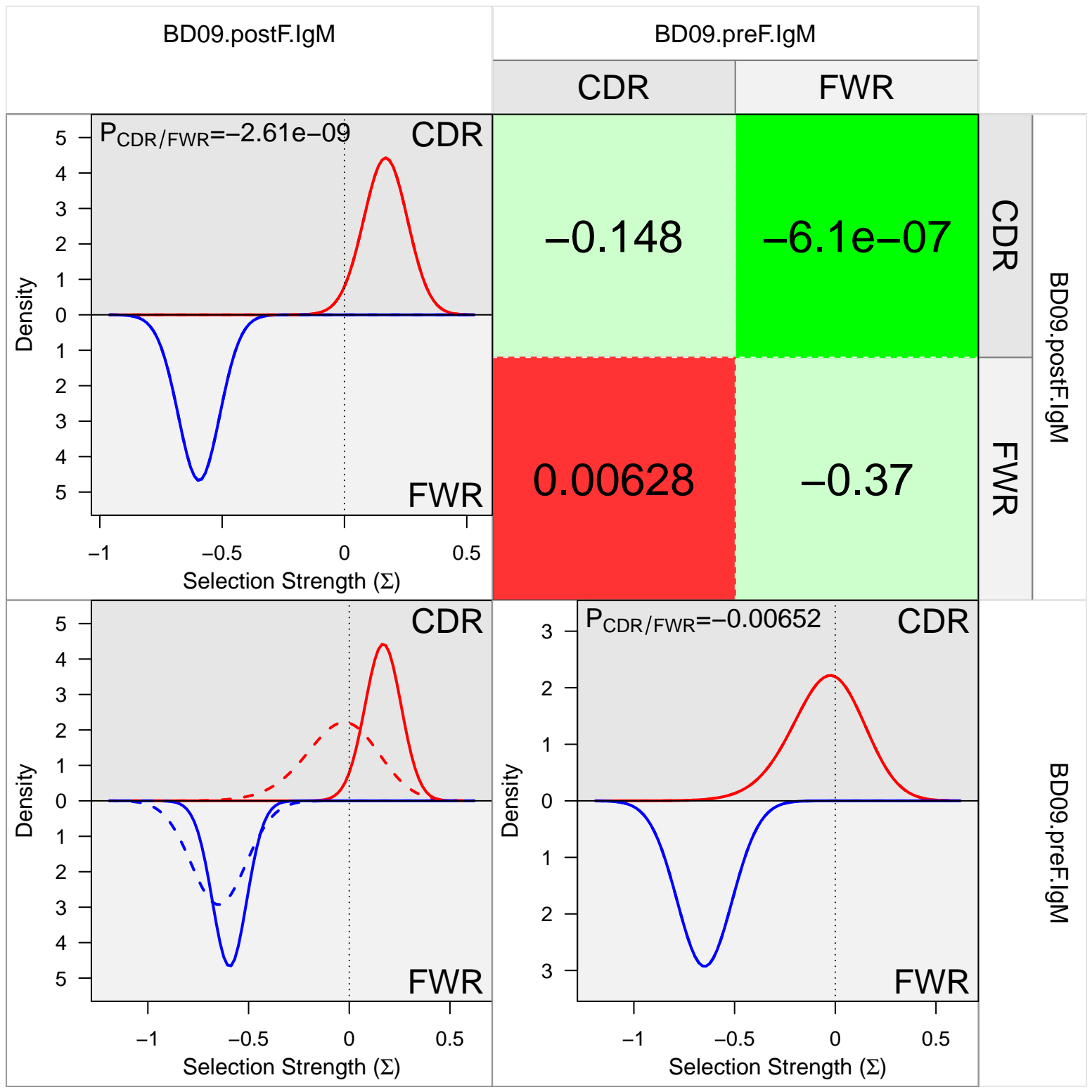

Supplement: Supplementary file 1 [file mmc1.zip › Supplementary_material/BD09.IgM.pdf]
